# Sclerosing mesenteritis: a benign cause of mesenteric mass lesions

**DOI:** 10.11604/pamj.2017.27.228.11542

**Published:** 2017-07-28

**Authors:** Diogo Carrola Gomes, Luísa Quaresma

**Affiliations:** 1 Surgery Service, Hospital Center Lisboa Central, Lisboa, Portugal

**Keywords:** Sclerosing mesenteritis, mesenteric panniculitis, mesenteric mass

## Abstract

Sclerosing mesenteritis is a rare disease of the mesentery. Associations with surgery, trauma, autoimmunity and paraneoplastic syndrome have been suggested, but most of the cases remain idiopathic. Diagnosis is often incidental, based upon the finding of a single or multiple mesenteric lesions on abdominal CT and histopathological confirmation. Optimal treatment is still controversial, but most of the cases reported have a favourable prognosis. We present a case of a 54-year-old male with long-standing abdominal pain and nausea, whose CT revealed the presence of a large mesenteric mass. A biopsy was performed, revealing benign chronic inflammation, fibrosis and IgG4-positive plasmocytes consistent with sclerosing mesenteritis. Clinical remission was achieved with corticosteroids and follow-up CTs at six and twelve months documented stability of the lesion. Furthermore, we review the current literature on the diagnosis and treatment options for this rare disease.

## Introduction

Sclerosing mesenteritis is a rare disease, part of a spectrum of idiopathic primary inflammatory and fibrotic processes that affect the mesentery [1]. It is often discovered incidentally, with a prevalence of 0.6% reported in the literature [2]. Clinical manifestations are varied and mostly unspecific, depending on the mass effect exerted by the lesions on the viscera and vessels. The most common complaint is abdominal pain, followed by abdominal distention, diarrhea and weight loss. Physical examination reveals a deep, poorly-defined abdominal mass in only about 50% of the patients [3]. It is therefore essential to maintain a high degree of suspicion for the differential diagnosis and ruling out malignancy. Diagnosis is attained from a combination of imaging and histopathologic findings. Dual-phase CT is the most sensitive imaging modality for detecting this disease. Two findings, "fat ring sign" and "tumor pseudocapsule" are considered specific for sclerosing mesenteritis [2, 3]. However, due to the broad differential diagnosis of mesenteric mass lesions, pathologic confirmation should be obtained in all suspected cases of sclerosing mesenteritis. We present this case report with the goal of raising awareness for this rare disease in patients with abdominal pain and mesenteric lesions.

## Patient and observation

**Figure 1:**
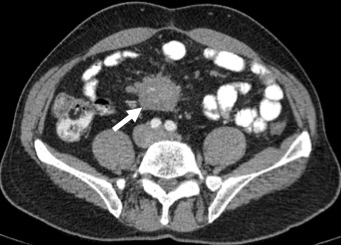
Axial CT scan with contrast, demonstrating a heterogenous soft tissue mass with calcifications and lymphadenopathy

**Figure 2:**
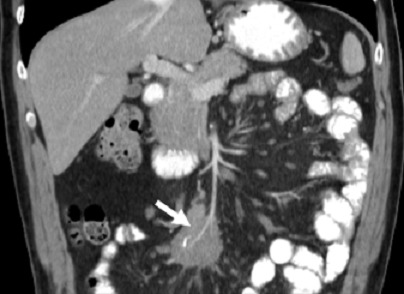
Coronal CT scan with contrast, demonstrating vascular encasement within the mesenteric lesion

**Figure 3:**
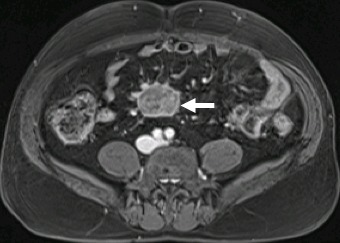
Axial T2 contrasted MRI, demonstrating delayed hyperenhancement and "tumor pseudocapsule"

**Figure 4:**
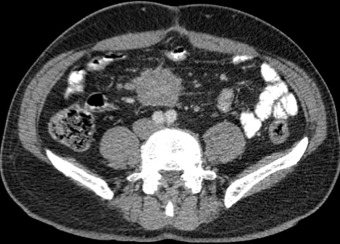
Axial CT scan with contrast 12 months after the diagnosis, showing stability of the lesion

A 54-year-old caucasian male with unremarkable medical history presented with worsening of a several-year history of lower-quadrant abdominal pain and nausea. There were no abnormal findings on his physical examination and laboratory workup. An abdominal CT revealed the presence of a soft tissue mass located in the root of the mesentery, 4.7x4.1x5cm, heterogenous with some calcifications within (Figure 1), with thickening of the surrounding fatty tissue, vascular encasement without infiltration of the vessels and lymphadenopathy (Figure 2). The lesion was further characterized by MRI as slightly hyperintense in T2 and hypointense in T1 with peripheral late phase contrast enhancement and "tumor pseudocapsule" (Figure 3). The patient was admitted for a surgical biopsy. During the surgery, we found a nodular thickening of the ileal mesentery, with apparent inflammation and retraction of the surrounding tissue. No other lesions were apparent. Since the size and location precluded a complete resection of the mass, several biopsies were performed and the fragments were sent for histopathologic review. The biopsy revealed a thickening of the connective tissue with fibrotic areas and lymphoplasmocytary infiltration rich in IgG4-positive plasmocytes. These findings were consistent with sclerosing mesenteritis. Serum IgG4 levels taken post-operatively were within the normal range. The patient was started on Prednisone (50mg a day) and quickly became asymptomatic. He was tapered off steroids after 3 months. Follow-up CTs at 6 and 12 months documented stability of the mesenteric mass (Figure 4).

## Discussion

The first case of sclerosing mesenteritis was reported by Jura in 1924 as "retractile mesenteritis" [4]. Since then, several other terms have been used to describe this entity, including mesenteric panniculitis, mesenteric lipodystrophy or mesenteric sclerosis. Emory et al [1] proposed that they represented different stages in the natural history of a single disease: firstly, adipocyte necrosis and infiltration of the mass by lipid-filled macrophages (mesenteric lypodistrophy); secondly, chronic inflammation with lymphocytic infiltrates and lipid necrosis (mesenteric panniculitis); finally, diffuse fibrosis with calcifications and shrinkage of the mesentery (sclerosing mesenteritis). The epidemiology is unknown. Daskalogiannaki et al reported a prevalence of 0.6% on abdominal CT examinations [2], but this number might be an underestimation. Most patients are Caucasian males in the fifth to seventh decades of life [1, 2]. The exact etiology of the inflammation and immune response that leads to this disease is still unknown. Associations with abdominal surgery and trauma, autoimmune processes, ischemic injury, malignancy and infections have been described [1, 3]. Symptoms are often unspecific, depending on the size and location of the mass and its relationship with the bowel, vessels and lymphatics [1, 3]. Akram et al [3] reported in a case series of ninety-two patients that the most common symptoms were abdominal pain (70%), bloating and distention (26%), diarrhea (25%) and weight loss (23%). Small bowel obstruction was present in 24% of patients and 10% were asymptomatic at the time of diagnosis. Physical examination reveals a deep, poorly defined abdominal mass in half of the patients, usually located in the left upper quadrant or epigastrium [1]. Blood tests results are often normal. The erythrocyte sedimentation rate and C-reactive protein level may be elevated and serve as markers of response to medical therapy [1]. The most common CT finding is a soft tissue mass in the small bowel mesentery. Kipfer et al [5] reported three patterns of lesions: type I with diffuse mesenteric thickening; type II with a single discrete mass; type III with multiple mass lesions. Types I and II appear to be the most common [3, 5]. Two CT signs are considered specific for SM: the "fat ring sign" is a preservation of the fatty tissue density around the mesenteric vessels and soft tissue lesions; "tumoral pseudocapsule" refers to the presence of a band of soft tissue surrounding the inflamed mass. Calcifications are present in about 20% of lesions, probably reflecting fat necrosis. Lymphadenopathy is present in 20-40% of patients [6].

MRI findings appear to be similar to CT, but a combination of the two has been suggested as having a higher sensitivity in the detection of sclerosing mesenteritis lesions [7]. The differential diagnosis includes all processes that can affect the mesentery. The most common radiological mimics are lymphoma, peritoneal carcinomatosis, carcinoid tumor, amyloidosis and mesenteric fibromatosis and mesenteric edema secondary to hypoalbuminemia, cirrhosis and congestive heart failure. Pathologic confirmation should be obtained in all cases of suspected SM to exclude malignancy. Since fine-needle aspiration specimens frequently lack enough architectural representation, a surgical biopsy is often needed. Some reports have shown that a subset of patients have lesions that stain abundantly for IgG4 and have proposed that this disease could be an IgG4-related sclerosing disorder. In the study by Akram et al [3], a third of the cases had abundant tissue infiltration by IgG4-positive plasma cells, while Chen et al [8] reported the same finding in two thirds of patients with sclerosing mesenteritis. Further studies are needed to assess the sensitivity and sensibility of IgG4 as a diagnostic marker for sclerosing mesenteritis [8]. An association between sclerosing mesenteritis and an elevated serum IgG4 and autoimmune pancreatitis has also been suggested [3]. Our patient, however, exhibited normal levels of serum IgG4 and had no personal or family history of autoimmune disorders. Due to the rarity of this disease, there is no recognized standard therapy regimen. Treatment is empiric and should be individualized. Immunomodulating agents such as glucocorticoids, colchicine, cyclophosphamide and tamoxifen have been reported to be beneficial in patients with non-obstructive symptoms. Complete surgical resection is often impossible due to disease extent and vascular compromise. Surgical bypass may be indicated to alleviate symptoms in case of small bowel obstruction [3, 9]. Although some cases of rapidly fatal disease have been reported largely due to complications of intestinal obstruction [3], sclerosing mesenteritis is believed to be a benign, stable or slowly progressing disease [10].

## Conclusion

Sclerosing mesenteritis is a rare disease, often diagnosed incidentally. Diagnosis is challenging due to the non-specificity of the symptoms and physical findings, requiring that physicians maintain a high level of suspicion for this disease when approaching patients with mesenteric lesions. Imaging with CT is the most sensitive diagnostic tool, but surgical biopsy is required to make the diagnosis and exclude radiological mimics. Treatment is mostly medical, with surgery being indicated in cases of severe obstruction. The disease course is believed to be benign and stable.

## Competing interests

The authors declare no competing interest.
